# PET quantification of brain *O*-GlcNAcase with [^18^F]LSN3316612 in healthy human volunteers

**DOI:** 10.1186/s13550-020-0616-4

**Published:** 2020-03-14

**Authors:** Jae-Hoon Lee, Jeih-San Liow, Soumen Paul, Cheryl L. Morse, Mohammad B. Haskali, Lester Manly, Sergey Shcherbinin, J. Craig Ruble, Nancy Kant, Emily C. Collins, Hugh N. Nuthall, Paolo Zanotti-Fregonara, Sami S. Zoghbi, Victor W. Pike, Robert B. Innis

**Affiliations:** 1 Molecular Imaging Branch, National Institute of Mental Health, National Institutes of Health, 10 Center Drive, Bethesda, MD 20892 USA; 2 Department of Nuclear Medicine, Yonsei University College of Medicine, Seoul, South Korea; 3 Molecular Imaging Core, University of Virginia, Charlottesville, VA USA; 4 The Centre for Molecular Imaging and Translational Research Laboratory, The Peter MacCallum Cancer Centre, Melbourne, Australia; 5 Eli Lilly and Company, Indianapolis, IN USA; 6 Houston Methodist Research Institute, Houston, TX USA

**Keywords:** *O*-GlcNAcase, Positron emission tomography, Tauopathy, Test-retest reliability, Neuroimaging

## Abstract

**Background:**

Previous studies found that [^18^F]LSN3316612 was a promising positron emission tomography (PET) radioligand for imaging *O*-GlcNAcase in nonhuman primates and human volunteers. This study sought to further evaluate the suitability of [^18^F]LSN3316612 for human clinical research.

**Methods:**

Kinetic evaluation of [^18^F]LSN3316612 was conducted in a combined set of baseline brain scans from 17 healthy human volunteers and test-retest imaging was conducted in 10 of these volunteers; another 6 volunteers had whole-body scans to measure radiation exposure to body organs. Total distribution volume (*V*_T_) estimates were compared for the one- and two-tissue compartment models with the arterial input function. Test-retest variability and reliability were evaluated via mean difference and intraclass correlation coefficient (ICC). The time stability of *V*_T_ was assessed down to a 30-min scan time. An alternative quantification method for [^18^F]LSN3316612 binding without blood was also investigated to assess the possibility of eliminating arterial sampling.

**Results:**

Brain uptake was generally high and could be quantified as *V*_T_ with excellent identifiability using the two-tissue compartment model. [^18^F]LSN3316612 exhibited good absolute test-retest variability (12.5%), but the arithmetic test-retest variability was far from 0 (11.3%), reflecting a near-uniform increase of *V*_T_ on the retest scan in nine of 10 volunteers. *V*_T_ values were stable after 110 min in all brain regions, suggesting that no radiometabolites accumulated in the brain. Measurements obtained using only brain activity (i.e., area under the curve (AUC) from 150–180 min) correlated strongly with regional *V*_T_ values during test-retest conditions (*R*^2^ = 0.84), exhibiting similar reliability to *V*_T_ (ICC = 0.68 vs. 0.64). Estimated radiation exposure for [^18^F]LSN3316612 PET was 20.5 ± 2.1 μSv/MBq, comparable to other ^18^F-labeled radioligands for brain imaging.

**Conclusions:**

[^18^F]LSN3316612 is an excellent PET radioligand for imaging *O*-GlcNAcase in the human brain. Alternative quantification without blood is possible, at least for within-subject repeat studies. However, the unexplained increase of *V*_T_ under retest conditions requires further investigation.

## Introduction

*O*-GlcNAcylation is a common post-transcriptional glycosylation that occurs extensively at the intracellular level of the brain [1]. This reversible cycling is modulated by two enzymes; *O*-linked β-*N*-acetylglucosamine (*O*-GlcNAc) transferase attaches *O*-GlcNAc to a protein, whereas *O*-GlcNAcase (OGA) removes it [2]. Since the discovery of *O*-GlcNAcylation of tau and its impact on tau phosphorylation, *O*-GlcNAcylation-related studies have rapidly increased in Alzheimer’s disease (AD) [3]. Notably, because hyperphosphorylated tau forms aggregates and neurofibrillary tangles—one of the hallmarks of AD [4]—*O*-GlcNAcylation and its relationship to phosphorylation have become the subjects of considerable investigative interest with regard to a group of neurodegenerative diseases collectively called tauopathies. *O*-GlcNAcylation has been known to stabilize microtubule-associated protein tau by hampering hyperphosphorylation and aggregation [5]. Indeed, initial ex vivo research supported the reciprocal relationship *O*-GlcNAcylation and phosphorylation [6], and analysis of human brain tissue revealed lower levels of tau-specific and overall *O*-GlcNAc in individuals with AD [7]. Furthermore, a series of animal studies found that increased *O*-GlcNAcylation cycling by an OGA inhibitor reduced tau protein aggregation and restored cognitive function [8–10]. Not surprisingly, rising levels of brain *O*-GlcNAcylation has been explored as a potential therapeutic strategy for attenuating the progression of AD and other tauopathies; as a result, potent and selective OGA inhibitors have been developed, and early phase clinical trials with these agents have begun [11, 12].

In this context, a positron emission tomography (PET) radioligand capable of imaging OGA could improve our understanding of the pathophysiology of neurodegenerative diseases, provide evidence of drug-target engagement, and help with dose selection of therapeutic candidates. Previous studies from our laboratory reported initial PET results of [^18^F]LSN3316612 (*N*-(5-(((2*S*,4*S*)-2-methyl-4-(6-fluoropyridin-2-yloxy) piperidin-1-yl)methyl)thiazol-2-yl)acetamide) in both nonhuman primates and healthy human volunteers. In nonhuman primates, [^18^F]LSN3316612 exhibited excellent properties for quantifying OGA in the brain, including high brain uptake with a high proportion of specific binding, lack of radiometabolite interference with quantification, and feasibility of quantification with compartmental modeling [13]. A follow-up study reported similarly promising results in the brains of eight healthy volunteers [14]; human brain uptake was well quantified with compartmental modeling and showed no evidence of accumulation of radiometabolites.

This study sought to further evaluate the suitability of [^18^F]LSN3316612 for use in human clinical research. Brain scans were evaluated from an additional set of volunteers (for a combined sample of 17). In addition, test-retest imaging was conducted in 10 volunteers, and 6 whole-body scans were conducted to measure radiation exposure to organs of the body.

## Material and methods

### Radioligand synthesis

[^18^F]LSN3316612 was synthesized by a nucleophilic substitution reaction on a nitro-aryl precursor, as previously described [13]. The nitro precursor (*N*-(5-(((2*S*,4*S*)-2-methyl-4-(6-nitropyridin-2-yloxy)piperidin-1-yl)methyl)thiazol-2-yl)acetamide) was provided at Eli Lilly.

### Participants

The entire population for both brain and whole-body imaging consisted of 11 male and 12 female healthy volunteers; 17 of these had brain scans and six had whole-body scans (Table 1). All volunteers were free of current medical or psychiatric illnesses as determined by medical history, physical examination, electrocardiogram, urinalysis, and laboratory blood tests (complete blood count, serum chemistries, and thyroid function test). The volunteer’s vital signs were recorded before radioligand injection and at 15, 30, 90, and 120 min post-injection.

**Table 1:** Demographic characteristics and PET scan parameters for 17 healthy volunteers injected with [^18^F]LSN3316612

Volunteer demographics and PET scan parameters	Kinetic evaluation (*n* = 17)	Test-retest study (*n* = 10)		Radiation dosimetry (*n* = 6)
	Baseline brain	Test	Retest	Whole body
Male:female (*n*)	8:9	5:5	5:5	3:3
Age (years)	40 ± 11	43 ± 11	43 ± 11	34 ± 16
Body weight (kg)	73 ± 17	72 ± 16	75 ± 17	69 ± 12
Injected activity (MBq)	187 ± 6	185 ± 7	189 ± 3	172 ± 47
Molar activity (MBq/nmol)	55 ± 22	45 ± 16	50 ± 13	64 ± 9
Injected mass dose (nmol/kg)	0.058 ± 0.033	0.069 ± 0.038	0.057 ± 0.016	0.040 ± 0.011

Data are presented as mean values (±SD)

### Brain image acquisition and processing

Brain PET scans were acquired from 17 healthy volunteers with an mCT scanner (Siemens Medical Solution, Cary, NC, USA). After a low-dose CT scan for attenuation correction, [^18^F]LSN3316612 (188 ± 5 MBq) was intravenously injected and PET data were acquired for 180 min with concurrent arterial blood sampling. Data were reconstructed into 45 frames (6 × 0.5 min, 3 × 1 min, 2 × 2 min, 34 × 5 min) using a three-dimensional ordered subset expectation-maximization algorithm. Brain uptake was expressed as a standardized uptake value (SUV), which normalizes for injected radioactivity and body weight. For structural magnetic resonance (MR) imaging, all participants underwent sagittal T1-weighted brain MR, using a 3T Philips Achieva scanner (Bothell, WA, USA) with turbo field echo sequence (repetition time = 8.1 ms, echo time = 3.7 ms, flip angle = 8, matrix = 181 × 256 × 256, voxel size = 1 × 0.983 × 0.983 mm).

Image pre-processing—such as coregistration between PET and MR, segmentation, and atlas normalization—was performed with the PNEURO pipeline implemented in PMOD 3.903 (Zurich, Switzerland). A total of 83 volumes of interest were defined based on the Hammers’ probabilistic brain atlas [15] and the subject’s individual MR image and subsequently combined into an individual template consisting of 16 regions that encompass the entire lobes of the brain and the principal subcortical structures: frontal, parietal, temporal, occipital, insula, amygdala, hippocampus, cingulate, striatum, thalamus, globus pallidus, brainstem, corpus callosum, cerebellar cortex, cerebellar white matter, and cerebral white matter. Regional time-activity curves were obtained by applying the template on the dynamic PET images transformed into MR space. The quality of PET-MR coregistration was visually assessed by a side-by-side comparison of PET, MR, and fused images at the end of PNEURO pipeline.

For the test-retest study, 10 out of the 17 volunteers who had a brain scan were scanned again on a different day under identical procedures. The interval between test and retest scans ranged from 8 to 150 days.

### Measuring [^18^F]LSN3316612 in plasma

During the brain PET scan, arterial blood was continuously monitored for 10 min at a rate of 5 mL/min, and radioactivity was measured with a cross-calibrated coincidence detector (PBS-101, Comecer, The Netherlands). Manual arterial samples were also obtained at 3, 5, 10, 15, 30, 60, 90, 120, 150, and 180 min after [^18^F]LSN3316612 injection.

For all blood samples, plasma concentrations of [^18^F]LSN3316612 were measured using an automatic gamma counter and were corrected after separation from radiometabolites using high-performance liquid chromatography (HPLC), as previously described [16], but with an X-Terra C18 column (10 μm, 7.8 × 300 mm; Waters Corp., Milford, MA, USA) and a mobile phase of MeOH:10 mM ammonium formate (75% by volume). The extraction efficiency of the deproteinization method (e.g., acetonitrile extraction) was quantified using the radioactivity of the precipitate. The mean extraction efficiency of the 10 blood samples obtained from each of the 10 participants during a 180-min test scan was 79.6% ± 6.4% (*N* = 10) which showed no significant difference from the 82.5% ± 5.7% mean that was obtained during the retest scans (*P* = 0.101). The plasma free fraction (*f*_P_; the non-protein bound fraction) was measured by ultrafiltration [16]. Using the blood-to-plasma ratios determined from the manual samples, total plasma radioactivity curves were obtained from measured whole blood data from automatic sampling for the first 10 min, then scaled to fit manual sample data. Total plasma radioactivity and whole blood activity were then fitted to a tri-exponential function. A Hill function [17] was used to fit the unchanged parent fraction. A time-activity curve of radiometabolite-corrected plasma parent radioactivity was generated implicitly by the product of the total plasma activity curve and parent fraction in PMOD, which was used as the input function.

### Tracer kinetic modeling

All kinetic analyses, including fitting blood curves, were conducted with the PKIN module in PMOD. The outcome measure, total distribution volume (*V*_T_), was calculated using one-and two-tissue compartmental models with noise equivalent count weighting and the radiometabolite-corrected arterial input function fitted to a tri-exponential function. Whole blood curves were used to correct for activity in the vascular component, assuming that blood volume was 5% of total brain volume. An optimal kinetic model was determined based on the relative fitness of the model (i.e., Akaike information criterion (AIC) and *F* test) and the identifiability of *V*_T_ (i.e., percent standard error (%SE) estimated from the theoretical parameter covariance matrix).

### Test-retest variability and reliability

Test-retest variability and absolute test-retest variability between test and retest scans were calculated as follows:

$$ \mathrm{Test}-\mathrm{retest}\ \mathrm{variability}\ \left(\%\right)=\frac{\mathrm{Test}\ \mathrm{value}-\mathrm{Retest}\ \mathrm{value}}{\left(\mathrm{Test}\ \mathrm{value}+\mathrm{Retest}\ \mathrm{value}\right)/2}\times 100 $$

$$ \mathrm{Absolute}\ \mathrm{test}-\mathrm{retest}\ \mathrm{variability}\ \left(\%\right)=\frac{\mid \mathrm{Test}\ \mathrm{value}-\mathrm{Retest}\ \mathrm{value}\mid }{\left(\mathrm{Test}\ \mathrm{value}+\mathrm{Retest}\ \mathrm{value}\right)/2}\times 100 $$

To assess test-retest reliability, the intra-class correlation coefficient (ICC) of each region was calculated as follows [18]:

$$ \mathrm{ICC}=\frac{\mathrm{BSMSS}-\mathrm{WSMSS}}{\mathrm{BSMSS}+\mathrm{WSMSS}} $$

where BSMSS and WSMSS are the mean sums of squares between subjects and within subjects, respectively. In the test-retest study, ICC value could range from – 1 to 1, and values closer to 1 indicated better reliability [19].

### Whole-body biodistribution and radiation dosimetry

To determine the radiation exposure to body organs, a separate group of six healthy volunteers underwent a whole-body PET scan after intravenous administration of [^18^F]LSN3316612 (172 ± 47 MBq). Dynamic scans were acquired using the same mCT scanner in seven contiguous segments from top of the head to mid-thigh in 14 frames of increasing duration (75 s to 15 min) for a total scan time of 120 min.

Thirteen source organs that could be identified as hot uptake foci on PET images were generously delineated on the tomographic images to ensure that all accumulated radioactivity in each organ was encompassed using PMOD: brain, heart, lungs, spleen, liver, kidneys, gallbladder, red marrow, stomach, testes/ovaries, urinary bladder, and small intestine. Uptake in the source organs was corrected with a recovery coefficient based on the average activity of the frames of the whole-body dynamic scan using large regions of interest drawn semi-automatically around the body. The average recovery coefficient of the 6 volunteers was 90%. The radioactivity concentration, measured without decay correction, was expressed as a percentage of injected dose for each organ. The organ residence time was calculated as the area under the time-activity curve using the trapezoid rule and physical decay after the last frame. Because the total red marrow present in the lumbar vertebrae accounts for ~ 12.3% of the mass of red marrow in the whole body [20], the red marrow final residence time was obtained by dividing the residence time in the lumbar vertebrae by 0.123. Values for the gastrointestinal tract were generated by the International Commission for Radiation Protection (ICRP) model in OLINDA/EXM1.1 [21] as activity entering the small intestine [22]. To obtain the residence time for the remainder of the body, residence times for all source organs were summed and then subtracted from the theoretical value of 2.65 h (=^18^F half-life/ln2).

### Statistical analysis

Quantitative results are presented as mean ± standard deviation (SD) unless otherwise noted. Differences between the test and retest groups were analyzed using a two-way *t* test, and those among the three groups of volunteers using the one-way analysis of variance (ANOVA). Bonferroni correction was used for multiple comparisons of each between-group comparison. The correlation between continuous variables was evaluated with linear regression analysis. Statistical significance was set at *P* < 0.05, and all statistical analyses were conducted using GraphPad Prism 5 (GraphPad Software, La Jolla, CA, USA).

## Results

### Study population and injection parameters

As noted above, 17 baseline scans were analyzed for kinetic evaluation of [^18^F]LSN3316612 in the brain; 7 volunteers had a single scan and 10 volunteers had test-retest scans. An arbitrary decision was made to use the first scan for those volunteers who had test-retest scans. Results for 8 of the 17 volunteers have previously been reported [14], and 1 of those volunteers went on to have a retest scan. A separate set of 6 volunteers underwent whole-body scans to evaluate biodistribution and radiation dosimetry. No significant differences were observed between the three groups of volunteers or between the test and retest studies with regard to injected activity, molar activity, and injected mass dose (Table 1).

### [^18^F]LSN3316612 uptake in brain

**Fig. 1:**
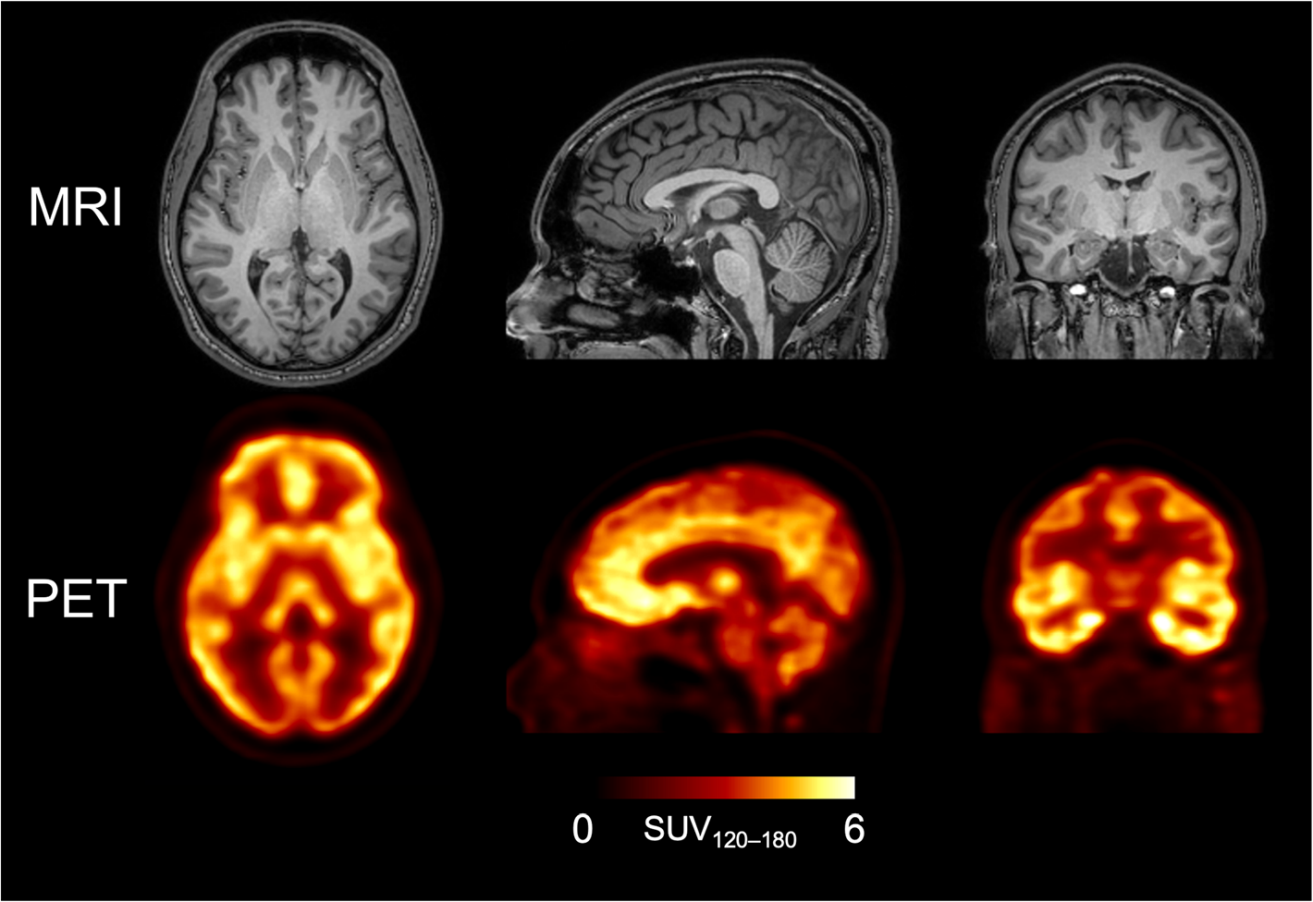
T1-weighted magnetic resonance (MR) and [^18^F]LSN3316612 positron emission tomography (PET) images of a healthy volunteer. Although the brain had high uptake of radioactivity, the skull had virtually none. The concentration of radioactivity in the brain is expressed as standardized uptake values (SUV), which was measured from 120 to 180 min

**Fig. 2:**
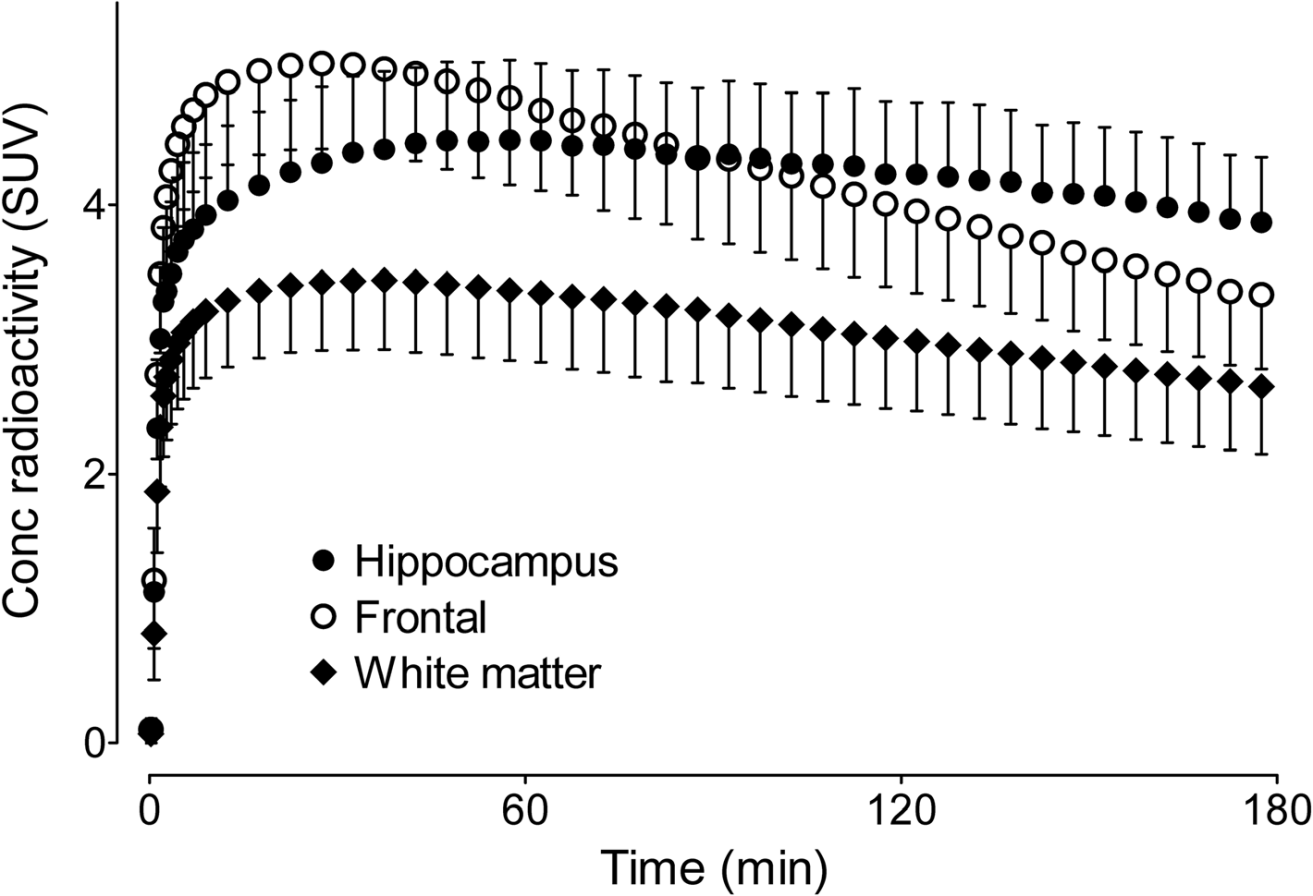
Time-activity curves for three brain regions after injection of [^18^F]LSN3316612. Points and bars represent mean standardized uptake values (SUV) and standard deviations (SD), respectively, which were measured from 17 healthy volunteers

After intravenous injection of [^18^F]LSN3316612, radioactivity in the brain increased rapidly, reached its maximum at 20–30 min with a mean peak SUV of ~ 5, and washed out slowly thereafter (Fig. 1). [^18^F]LSN3316612 showed widespread and moderately high uptake in the brain preferentially along grey matter regions (Fig. 2). The highest radioactivity concentrations (SUV) were observed in the frontal cortex (~ 5.1), followed by the striatum and occipital cortex (~ 5.0). The lowest radioactivity concentrations were observed in the corpus callosum (~ 2.3), brainstem (~ 3.3), and cerebral white matter (~ 3.5). There was almost no uptake of radioactivity in the skull, suggesting that little, if any, radiodefluorination occurred.

### Metabolism and clearance of radioligand in plasma

**Fig. 3:**
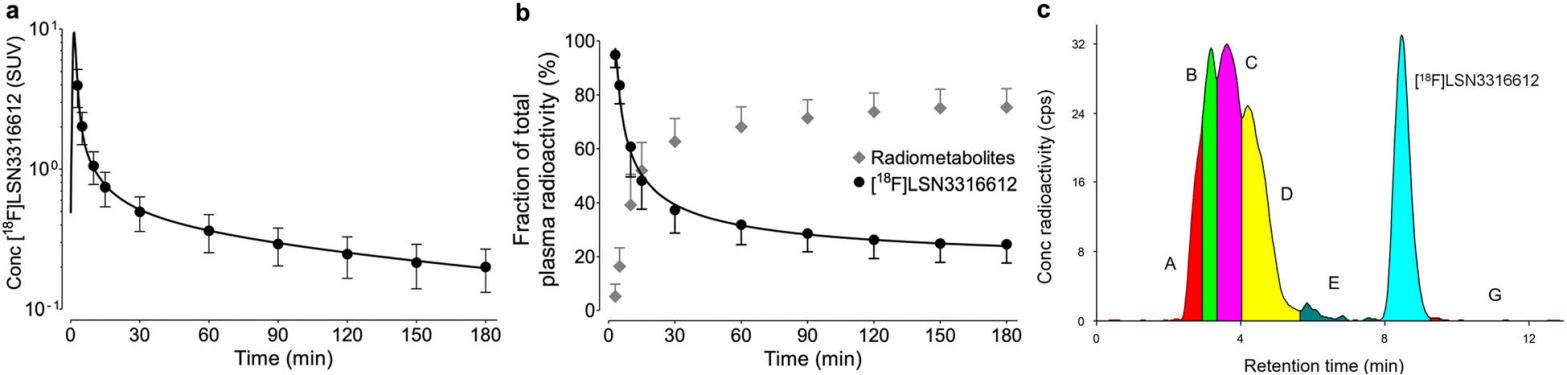
Parent radioligand and radiometabolite profile in plasma after injection of [^18^F]LSN3316612. **a** The mean concentration (±SD) of [^18^F]LSN3316612 in plasma from 17 healthy volunteers. **b** The fractions (±SD) of parent radioligand and radiometabolites in plasma from 17 healthy volunteers. **c** High-performance liquid chromatography (HPLC) radiochromatogram of plasma content at 120 min after injection of [^18^F]LSN3316612 in one healthy volunteer

After an initial peak at about 1.5 min, the concentration of parent radioligand in plasma decreased rapidly and was well fitted to a triexponential curve (Fig. 3a). The concentration of parent radioligand was equal to that of all radiometabolites at about 15 min (Fig. 3b). The HPLC radiochromatogram of plasma at 120 min identified at least five radiometabolites, all of which were less lipophilic than the parent radioligand (Fig. 3c). The *f*_P_ of parent radioligand was 4.2 ± 1.1%.

### Tracer kinetic evaluation of [^18^F]LSN3316612 in brain

**Fig. 4:**
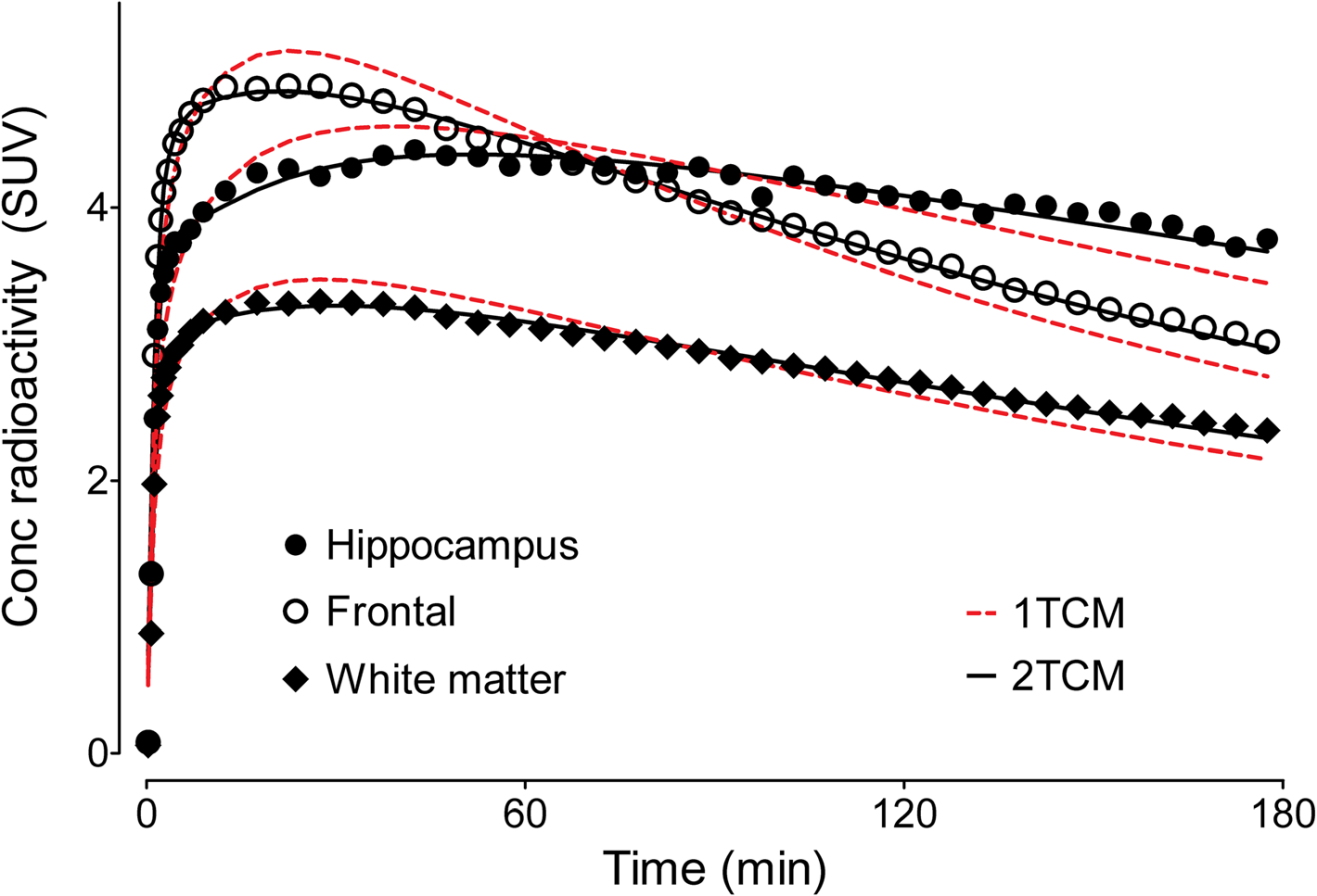
Pharmacokinetic fitting of brain time-activity curves using one- and two-tissue compartment models. The two-tissue (2TCM, black lines) compartment model better fit the measured standardized uptake values (SUV) than the one-tissue (1TCM, red dotted line) compartment model, both visually and statistically

Using serial measurements of radioactivity in the brain and the concentration of parent radioligand in arterial plasma, regional OGA density was well quantified as *V*_T_ using a two-tissue compartment model. Compared to the one-tissue compartment model, the two-tissue compartment fit the data better both on visual inspection (Fig. 4) and statistical comparison. That is, the AIC was lower in the two-tissue compartment model than in the one-tissue compartment model (545.5 and 640.7, respectively; *P* = 0.004), and *F* tests indicated a significantly better fit for the two-tissue compartment model. These results are consistent with the radioligand having two kinetically distinct binding sites: a nonspecific binding site with low affinity and rapid equilibration, and a specific binding site with high affinity and slow equilibration.

Using the two-tissue compartment model, the mean regional *V*_T_ (mL·cm^−3^) was 12.2 and ranged from 7.9 to 17.6 (Table 2). The identifiability of *V*_T_ was excellent, with standard error (SE) less than 10% for all brain regions. The three brain regions with the highest *V*_T_ were the amygdala (17.6), hippocampus (15.5), and cingulate region (14.5), and the three regions with the lowest *V*_T_ were brainstem (7.9), corpus callosum (8.0), and white matter (9.1) (Table 2). The estimated rate constants of the whole brain were 0.301 ± 0.155 mL·min^−1^·mL^−1^ for *K*_1_, 0.113 ± 0.076 min^−1^ for *k*_2_, 0.092 ± 0.061 min^−1^ for *k*_3_, and 0.026 ± 0.007 min^−1^ for *k*_4_, respectively. The identifiability of the rate constants was excellent; the mean SE was less than 10% (range 1.8–8.6%) for *K*_1_, *k*_2_, and *k*_4_, and it was 11.6% for *k*_3_. The *V*_T_ of the whole brain did not significantly differ between men and women and did not correlate with age. However, the sample size was small (*n* = 17), and the age range was limited (23–57 years old).

**Table 2:** Total distribution volume (*V*_T_) of brain regions after injection of [^18^F]LSN3316612

Region	*V*_T_ (mL·cm^−3^)^a^	
	Mean	COV (%)
Amygdala	17.6	28.3
Hippocampus	15.5	24.6
Cingulate	14.5	25.2
Insula	14.4	24.0
Temporal	13.9	24.2
Frontal	12.9	23.8
Striatum	12.7	23.7
Parietal	12.2	22.2
Occipital	11.6	21.6
Globus pallidus	11.5	25.0
Cerebellum	11.3	22.3
Thalamus	11.2	22.8
White matter (WM)	10.3	26.2
Cerebellar WM	9.1	23.7
Corpus callosum	8.0	36.8
Brainstem	7.9	24.5
Mean	12.2	24.9

*COV* coefficient of variation

^a^*V*_T_ was calculated using a two-tissue compartment model. Values represent the mean and coefficient of variation (COV = mean/SD) from the baseline scans of 17 healthy volunteers

### Test-retest variability and reliability

Variability of *V*_T_ for the test-retest scans was good. However, reliability was only modest, in part because the retest *V*_T_ of the whole brain was higher than that of the test scan in 9 out of 10 volunteers (Additional file 1: Table S1), with the mean value increased by 12.1% (*P* = 0.012). Test-retest variability and absolute test-retest variability were 11.3% and 12.5%, respectively, across all brain regions. The mean ICC was 0.64. In addition, ICC was relatively poor for the amygdala (0.42) and hippocampus (0.41), which were the regions with highest *V*_T._

With regard to test-retest scans, the interval between the two scans was 49 ± 47 days (range 8–150 days). Investigation of the interval between test and retest, seasonal or diurnal variations, molar activity, injected mass dose of the test scan, and *f*_P_ found that none of these factors correlated with the increase in *V*_T_ (Additional file 2: Table S2). Although plasma glucose was 13.6% lower on retest compared to test scans, the significance (*P* = 0.018) did not survive multiple comparison tests.

### Time stability of *V*_T_

**Fig. 5:**
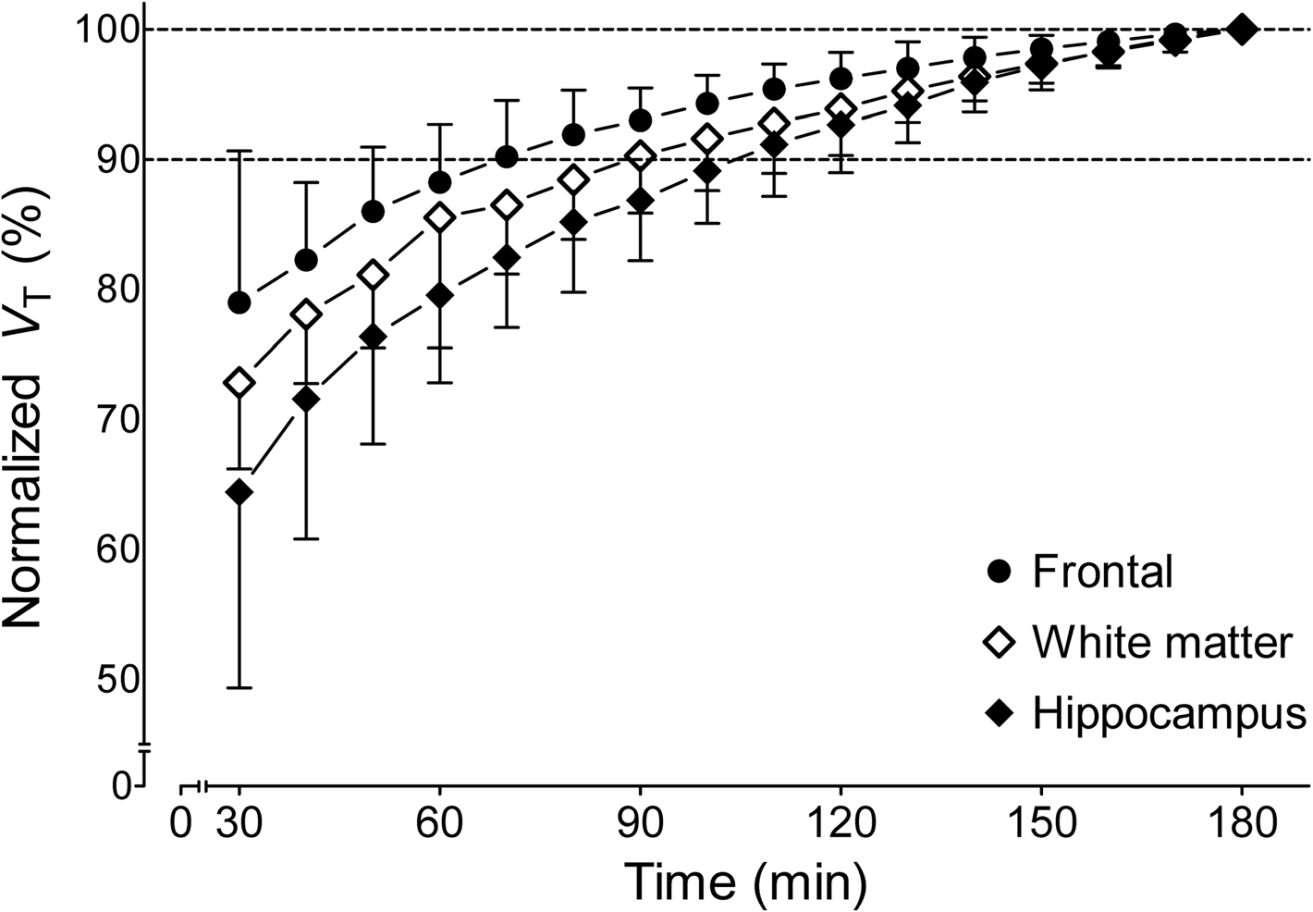
Time stability analysis of the total distribution volume (*V*_T_) of radioactivity within three brain regions. *V*_T_ was calculated via a two-tissue compartment model and normalized to the terminal *V*_T_ value at 180 min. Points represent the mean normalized *V*_T_ (±SD) from 17 healthy volunteers

The time stability of *V*_T_ was evaluated by fitting the PET data with truncated acquisition times ranging from 0–30 to 0–180 min; these were expressed as a percentage of the value determined with the entire 180-min scan. Regional *V*_T_ value asymptotically reached the terminal value of *V*_T_ (Fig. 5). For all brain regions, the average *V*_T_ values converged within 10% of their terminal values by 110 min. The hippocampus was the slowest region to reach the stable *V*_T_ value with 91% of the terminal value at 110 min, while the cerebellum was the fastest, with 92% in only 60 min. The relatively stable *V*_T_ values for the last 70–120 min are consistent with the lack of accumulation of radiometabolites in the brain. That is, uptake and washout of brain radioactivity could be explained by the input of only parent radioligand.

### Alternative quantification of [^18^F]LSN3316612 binding

**Fig. 6:**
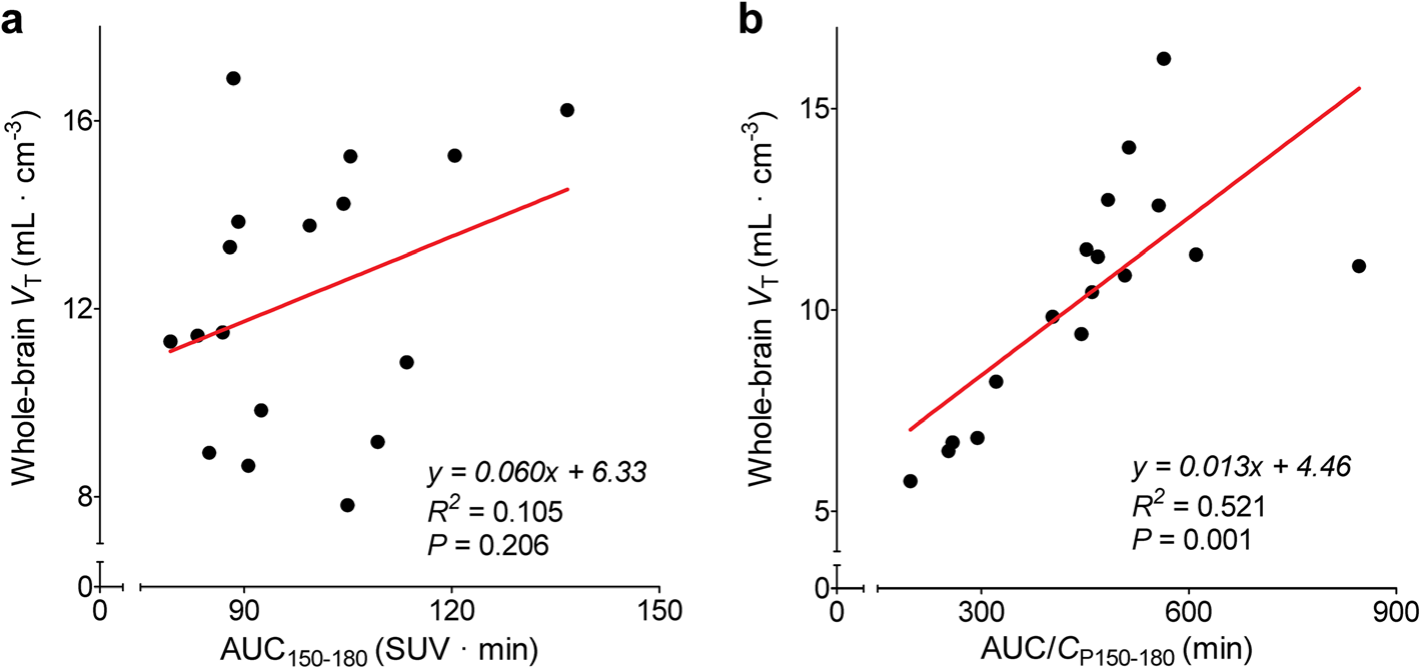
Correlation of the “gold-standard” measurement of whole-brain total distribution volume (*V*_T_) and two more simplified values. **a** Area under the brain time-activity curves measured from 150 to 180 min (AUC_150–180_). **b** The same value normalized (i.e., divided) by the mean concentration of parent radioligand in arterial plasma measured at 150 and 180 min (AUC/*C*_P150–180_). The points represent individual values from 17 healthy volunteers. The line represents a linear fit of the points

Because it uses both brain and plasma concentrations of radioactivity, *V*_T_ is the ‘gold standard’ against which simplified approaches are compared. For this study, three potential ways of avoiding arterial blood sampling, thus simplifying the experimental methods, were considered. The first and arguably best approach would have been to use a reference region that contained no receptors. However, blocking studies in monkeys showed that all brain regions, including white matter, had displaceable uptake [13]. Second, we considered whether brain uptake by itself might be able to substitute for measures like *V*_T_. We found that among three 30-min time intervals (150–180 min, 120–150 min, and 90–120 min), the AUC of 150–180 min (AUC_150–180_) correlated best with regional *V*_T_ calculated using arterial input function (*R*^2^ 0.84, 0.81, and 0.74, respectively). However, although AUC_150–180_ correlated strongly with *V*_T_ on test and retest scans in an individual volunteer, it correlated poorly with *V*_T_ when tested across all 17 volunteers (Fig. 6a). Third, we considered whether a few blood samples could substitute for the entire input function by normalizing brain activity to the concentration of parent radioligand in arterial plasma—i.e., AUC/*C*_P150–180_. This normalization slightly improved the correlation with *V*_T_ across all 17 volunteers (Fig. 6b), but the reliability in test-retest scans of AUC/*C*_P150–180_ (ICC = 0.18) was much worse than that of AUC_150–180_ by itself (ICC = 0.68; Table 3). In summary, none of these three simplifications were useful in comparing OGA densities between individuals. However, brain activity by itself (AUC_150–180_) might be used for repeated studies in a single volunteer (e.g., to measure enzyme occupancy by a therapeutic candidate) but would need to be corrected for the consistent trend of the retest scan being higher than the test scan.

**Table 3:** Variability and reliability of test-retest parameters from 10 healthy volunteers after injection of [^18^F]LSN3316612

Parameter^a^	Source	Test^b^	Retest^b^	TRV (%)	aTRV (%)	ICC
*V*_T_	Brain and plasma	13.7 (19.2)	15.3 (19.6)	11.3	12.5	0.64
AUC_150–180_	Brain	102.2 (16.3)	103.6 (11.9)	1.8	9.0	0.68
AUC/*C*_P150–180_	Brain and plasma	654.1 (46.2)	661.1 (19.7)	6.5	31.9	0.18
Plasma parent AUC_0–∞_	Plasma	105.9 (20.3)	96.0 (20.3)	− 9.7	12.3	0.69
Brain AUC_0–∞_	Brain	1428.2 (23.3)	1445.5 (20.6)	1.6	13.3	0.78

*TRV* test-retest variability, *aTRV* absolute test-retest variability, *ICC* intraclass correlation coefficient, *V*_T_ total distribution volume, *AUC* area under the curve, *TAC* time-activity curve

^a^The measured parameters were *V*_T_ total distribution volume (mL·cm^−3^); AUC_150–180_ area under the curve of brain TAC of 150–180 min; AUC/C_P150–180_ normalized AUC_150–180_ to mean parent radioactivity concentration in arterial plasma; AUC_0–∞_ area under the curve estimated from 0 min to infinity

^b^Data are presented as mean with coefficient of variation (%) in parentheses

### Whole-body biodistribution and radiation dosimetry

**Fig. 7:**
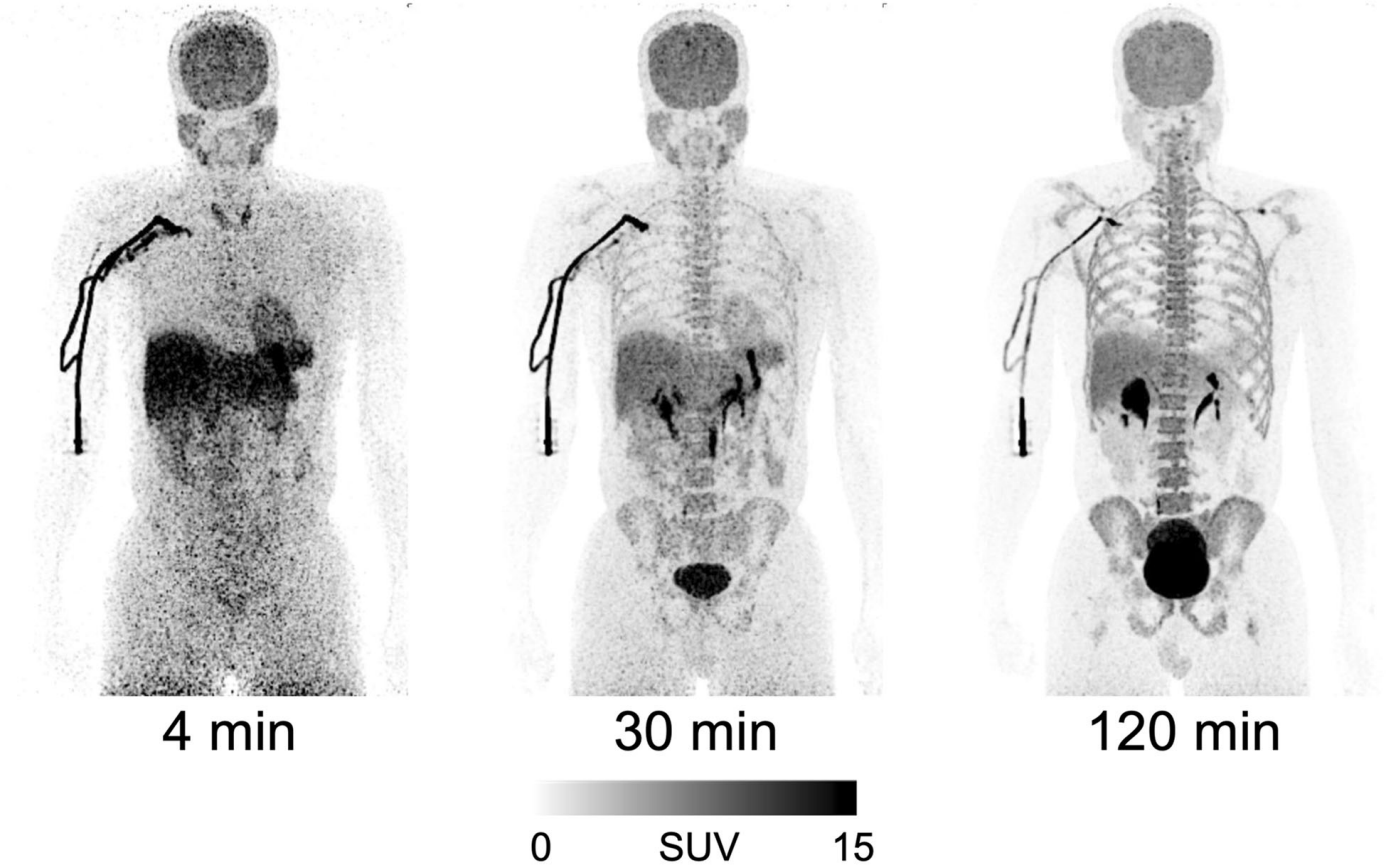
Whole-body images of a healthy male at 4, 30, and 120 min after [^18^F]LSN3316612 injection. The images are displayed in standardized uptake value (SUV) as a two-dimensional compression with maximal intensity projected (MIP) forward to emphasize contrast at the edge of organs. The images are decay-corrected to time of injection

Whole-body images were notable for early distribution in the blood pool, accumulation in the target organ (i.e., brain), and excretion via urinary tract (Fig. 7). The brain had high radioactivity concentration, with a peak of about 6% of the injective dose at 30 to 40 min post-injection. The liver and brain had the longest residence times (0.166 ± 0.008 and 0.160 ± 0.011 h, respectively). The three organs with the highest exposure (μSv/MBq) were the urinary bladder (86.4), brain (32.2), and liver (30.4) (Additional file 3: Table S3). The mean effective dose derived from six healthy volunteers was 20.5 ± 2.1 μSv/MBq. Thus, injection of 185 MBq of [^18^F]LSN3316612 would lead to an effective dose of 3.8 mSv; this dose is similar to that of other ^18^F-labeled radioligands used for brain imaging [23].

## Discussion

The present study found that [^18^F]LSN3316612 was an excellent PET radioligand for quantifying OGA in the human brain with the exception of an unexplained increase in *V*_T_ on retest scanning in nine of 10 volunteers. Brain uptake was generally high and could be quantified as *V*_*T*_ with excellent identifiability using a two-tissue compartment model. [^18^F]LSN3316612 exhibited good absolute test-retest variability (~ 12.5%), but the arithmetic test-retest variability was far from 0 (~ 11.3%), reflecting an almost uniform increase of *V*_T_ on retest scanning. *V*_*T*_ values were stable after 110 min of scanning in all regions, suggesting that radiometabolites did not accumulate in the brain. Efforts to investigate an alternative quantification method for [^18^F]LSN3316612 binding without blood to assess the possibility of eliminating arterial sampling found that measurements obtained using only brain activity (i.e., AUC from 150 to 180 min) were strongly correlated with regional *V*_T_ values within an individual volunteer during test and retest conditions (*R*^2^ = 0.84), and similar reliability to *V*_T_ was observed (ICC = 0.68 for AUC_150–180_ and 0.64 for *V*_T_).

Despite the promising characteristics of [^18^F]LSN3316612, the unexplained increase of *V*_T_ under retest conditions requires further investigation before the radioligand can be widely used. In this study, the “gold standard” measurement of OGA density (i.e., *V*_T_) in retest scans was greater than that in the test scans in 9 out of 10 volunteers, with an average increase of 12.1%. This unexpected increase may reflect two possibilities. The first is that OGA levels actually increased on retest scans, and the second is that our measurement of *V*_T_ was flawed. With regard to the first possibility, several factors were examined that might correlate with and thereby explain an increase in retest scan. These included interval between test and retest, seasonal or diurnal variations, molar activity, injected mass dose of the test scan, *f*_P_, image acquisition and processing, and the volunteers’ laboratory test results (Additional file 2: Table S2). During the entire course of the study, factors such as variation of dose calibrator, well counter, and HPLC were all closely monitored, and we found no significant change in the external detectors related to radioactivity measurement. Therefore, none of these factors, we believe, contributed to the increase of *V*_T_. Nevertheless, the sample size was small (*n* = 10) and may have had inadequate power to detect true effects.

The second, and more likely, the possibility is that our measurement of *V*_T_ was flawed in some way. Indeed, the data suggested potential errors in measuring the input function, i.e., the concentration of parent radioligand in plasma over time. In particular, the variability and reliability of only brain activity (i.e., AUC_150–180_) was as good as or slightly better than that of *V*_T_, which uses brain and plasma data. Addition of mean parent concentration in plasma during the last 30 min (i.e., AUC/*C*_P150–180_) worsened the variability and reliability in comparison to only AUC_150–180_. To explore whether measurement of the input function was flawed, we measured the AUC from time zero to infinity (AUC_0–∞_) of the parent radioligand in plasma to determine how it may have affected *V*_T_. Please note that *V*_T_ = AUC_0–∞_ of brain curve/AUC_0–∞_ of the plasma curve. We found that the AUC_0–∞_ of the plasma curve was 8.6% lower on retest compared to test scans, which would explain most of the increase in *V*_T_ (12.1%). To determine which component of the plasma curve might have been measured inaccurately, we explored tri-exponential fitting of the plasma curve, which identified three half-lives (1.0 ± 0.4, 8.0 ± 3.3, and 140.7 ± 39.2 min, respectively). The third (slowest) component contributed most (76.3%) of the AUC_0–∞_. Thus, sampling for only 180 min might inadequately define an exponent with a half-life of 141 min. In addition, the concentration of radioactivity in plasma was decreasing and difficult to measure. As a result, we suspect, but are not certain, that errors may have occurred in the measurement of the plasma input function to increase *V*_T_ on retest scans. However, we cannot explain why a consistent bias would have existed so that plasma AUC_0–∞_would be lower on retest. A study with a larger sample size and longer plasma sampling might help answer these questions.

The average *V*_T_ values in the 17 volunteers who had brain scans reached 90% of terminal 3-h values at 110 min and remained stable thereafter. This indicates that no troublesome radiometabolites entered the brain. Regions with high *V*_T_ (e.g., hippocampus) were slower to reach stable *V*_T_ than regions with lower *V*_T_ (e.g., cerebellum), consistent with the notion that higher enzyme density regions require a longer time to reach equilibrium. The evaluation of time stability revealed that the [^18^F]LSN3316612 PET scan time could be reliably shortened to 120 min; however, time stability would need to be re-evaluated within disease-specific groups because the equilibrium time might change depending on the degree of reduced OGA expression and decreased blood flow that can occur in tauopathies.

Following the injection of [^18^F]LSN3316612, the whole-body distribution of radioactivity reflected both the distribution of OGA and the metabolism of the radioligand. OGA is expressed in relatively high density in the brain, gastrointestinal tract, exocrine glands, and immune system [24, 25]. Accordingly, whole-body [^18^F]LSN3316612 PET exhibited high accumulation in the brain, small bowel, salivary glands, and spleen. Similarly, uptake in the lumbar vertebrae, which contain approximately 12.3% of all red marrow in the body [20], likely reflected uptake in hematopoietic stem cells in the red marrow [25]. Most of the radioactivity was in the bone marrow and not in distal appendicular bones without red marrow, suggesting that negligible defluorination, if any, occurred. In addition to reflecting the distribution of OGA in the body, radioactivity also likely reflected metabolism due to the hepatobiliary tract excretion of radiometabolites via urine—e.g., a glucuronidated radiometabolite of the lipophilic radioligand. The urinary bladder also received relatively high exposure, although this was probably due to long retention of urine during the 3-h PET scan and could therefore be modestly lowered during a typical 2-h scan and by immediate voiding after completion of PET scan. The effective dose (μSv/MBq) of [^18^F]LSN3316612 (20.5 ± 2.1) was similar to that of other ^18^F-radioligands used for brain imaging (20.8 ± 6.7 for 21 radioligands) [23].

## Conclusions

Taken together, our results suggest that [^18^F]LSN3316612 is an excellent radioligand for use in clinical research. This ligand shows promise for elucidating the pathophysiology of neurogenerative diseases and facilitating drug development by providing in vivo measures of target engagement and enzyme occupancy. However, further studies are needed to fully understand the unexplained increase in *V*_T_ under retest conditions before the radioligand can be widely used.

## Supplementary information

**Additional file 1: Table S1.** Brain region test-retest variability and reliability for total distribution volume (*V*_T_) measurements in 10 healthy volunteers injected with [^18^F]LSN3316612.

**Additional file 2: Table S2.** Demographic and imaging variables for the 10 healthy volunteers in the test-retest study.

**Additional file 3: Table S3.** Radiation dose estimates from six healthy volunteers injected with [^18^F]LSN3316612.

## Data Availability

The datasets used in this study are available from the corresponding author on reasonable request.

## Abbreviations

AD: Alzheimer’s disease
AIC: Akaike information criterion
AUC: Area under the curve
*f*_P_: Plasma-free fraction
HPLC: High-performance liquid chromatography
ICC: Intraclass correlation coefficient
ICRP: International Commission for Radiation Protection
OGA: *O*-GlcNAcase
*O*-GlcNAc: *O*-linked β-*N*-acetylglucosamine
PET: Positron emission tomography
SD: Standard deviation
SE: Standard error
*V*_T_: Total distribution volume
