# Supplementary material for: PET quantification of brain O-GlcNAcase with [18F]LSN3316612 in healthy human volunteers
Source: EJNMMI Res. 2020 Mar 14;10:20. doi: 10.1186/s13550-020-0616-4 (PMC7072082; doi:10.1186/s13550-020-0616-4)
Supplement: Supplementary file 1 — Additional file 1: Table S1. Brain region test-retest variability and reliability for total distribution volume (VT) measurements in 10 healthy volunteers injected with [18F]LSN3316612. [file 13550_2020_616_MOESM1_ESM.docx]

**Table S1.** Brain region test-retest variability and reliability for total distribution volume (*V*_T_) measurements in 10 healthy volunteers injected with [^18^F]LSN3316612.

| Brain region^a^ | *V*_T_ (mL·cm^-3^)^b^ | | TRV (%) | aTRV (%) | ICC |
| --- | --- | --- | --- | --- | --- |
|  | Test | Retest |  |  |  |
| Amygdala | 20.0 (21.8) | 23.4 (23.5) | 15.8 | 18.2 | 0.42 |
| Hippocampus | 17.5 (17.4) | 20.0 (18.7) | 12.8 | 13.4 | 0.41 |
| Cingulate | 16.2 (19.4) | 18.2 (19.1) | 11.5 | 12.4 | 0.67 |
| Insula | 16.1 (17.7) | 17.9 (17.1) | 10.8 | 11.9 | 0.66 |
| Temporal | 15.6 (18.3) | 17.5 (18.7) | 11.9 | 12.8 | 0.62 |
| Frontal | 14.6 (17.5) | 16.2 (17.6) | 10.9 | 11.5 | 0.68 |
| Striatum | 14.4 (15.6) | 16.2 (18.0) | 11.2 | 11.9 | 0.55 |
| Parietal | 13.7 (16.2) | 15.0 (16.9) | 9.2 | 11.2 | 0.67 |
| Globus pallidus | 13.1 (17.6) | 14.9 (24.1) | 11.7 | 12.8 | 0.61 |
| Occipital | 12.9 (16.5) | 14.2 (17.3) | 9.6 | 10.4 | 0.70 |
| Thalamus | 12.6 (16.2) | 13.9 (19.4) | 10.1 | 10.8 | 0.65 |
| Cerebellum | 12.4 (18.3) | 13.9 (16.8) | 11.5 | 12.7 | 0.63 |
| White matter (WM) | 11.6 (20.7) | 12.8 (22.0) | 9.4 | 10.1 | 0.80 |
| Cerebellar WM | 10.1 (18.5) | 11.3 (19.5) | 11.3 | 12.8 | 0.65 |
| Corpus callosum | 9.0 (35.0) | 9.7 (23.0) | 9.7 | 12.3 | 0.86 |
| Brainstem | 8.8 (19.7) | 10.1 (21.3) | 13.5 | 14.8 | 0.59 |
| Average | 13.7 (19.2) | 15.3 (19.6) | 11.3 | 12.5 | 0.64 |

TRV: test-retest variability; aTRV: absolute test-retest variability; ICC: intraclass correlation coefficient; *V*_T_: total distribution volume.

^a^ Regions are listed in order of *V*_T_, from highest to lowest. Test-retest reliability tended to be worse in those regions with higher *V*_T_.

^b^ Data are presented as mean with coefficient of variation (%) in parenthesis. *V*_T_ was derived with a two-tissue compartment model.
