# Supplementary material for: PET quantification of brain O-GlcNAcase with [18F]LSN3316612 in healthy human volunteers
Source: EJNMMI Res. 2020 Mar 14;10:20. doi: 10.1186/s13550-020-0616-4 (PMC7072082; doi:10.1186/s13550-020-0616-4)
Supplement: Supplementary file 2 — Additional file 2: Table S2. Demographic and imaging variables for the 10 healthy volunteers in the test-retest study. [file 13550_2020_616_MOESM2_ESM.docx]

**Table S2.** Demographic and imaging variables for the 10 healthy volunteers in the test-retest study.

| Variables | Test^a^ | Retest^a^ | *P* value^b^ |
| --- | --- | --- | --- |
| Weight (kg) | 72.2 ± 16.1 | 74.8 ± 16.6 | 0.256 |
| Body surface area (m^2^) | 1.84 ± 0.25 | 1.87 ± 0.26 | 0.275 |
| Injected activity (MBq) | 185.2 ± 6.9 | 188.7 ± 3.4 | 0.239 |
| Molar activity (MBq/nmol) | 45.2 ± 15.8 | 50.0 ± 12.9 | 0.525 |
| Injected mass dose (nmol/kg) | 0.069 ± 0.038 | 0.057 ± 0.016 | 0.331 |
| *f*_P_ | 0.040 ± 0.012 | 0.037 ± 0.008 | 0.496 |
| Age *vs.*  test-retest variability^c^ |  |  | 0.549 |
| Interval between test and retest *vs.*  test-retest variability^c^ |  |  | 0.142 |
| Scan season^d^ | Spring (60%)  Summer (20%)  Winter (20%) | Spring (60%)  Summer (30%)  Winter (10%) | 0.850 |
| Scan start time | 11:20 AM | 11:01 AM | 0.182 |
| Glucose (mg/dL)^e^ | 93.3 ± 15.2 | 80.6 ± 6.2 | 0.018 |
| Albumin (g/dL) | 4.17 ± 0.34 | 4.13 ± 0.29 | 0.779 |
| Protein (g/dL) | 7.17 ± 0.33 | 7.06 ± 0.71 | 0.630 |

*f*_P:_ plasma free fraction

^a^ Test and retest values are presented as mean (± SD) or frequency.

^b^ The reported *P* values are not corrected for multiple comparisons. If they had been, the change in glucose (*P* = 0.018) would not be significant (*P* < 0.05).

^c^ Linear regression was performed to evaluate the correlation between two variables.

^d^ Fisher’s exact test was performed to evaluate the difference in proportion between groups.

^e^ No significant group difference was found in the other blood test results.
