# Supplementary material for: PET quantification of brain O-GlcNAcase with [18F]LSN3316612 in healthy human volunteers
Source: EJNMMI Res. 2020 Mar 14;10:20. doi: 10.1186/s13550-020-0616-4 (PMC7072082; doi:10.1186/s13550-020-0616-4)
Supplement: Supplementary file 3 — Additional file 3: Table S3. Radiation dose estimates from six healthy volunteers injected with [18F]LSN3316612. [file 13550_2020_616_MOESM3_ESM.docx]

**Table S3.** Radiation dose estimates from six healthy volunteers injected with [^18^F]LSN3316612.

| Organ | Estimated radiation dose  (µSv/MBq) |
| --- | --- |
| Urinary bladder | 86.4 ± 22.2 |
| Brain | 32.2 ± 6.7 |
| Liver | 30.4 ± 4.7 |
| Kidneys | 29.9 ± 4.3 |
| Gallbladder | 25.4 ± 6.3 |
| Small intestine | 25.4 ± 3.0 |
| Spleen | 24.4 ± 8.5 |
| Osteogenic cells | 24.1 ± 3.7 |
| Stomach | 20.3 ± 3.7 |
| Red marrow | 19.1 ± 2.5 |
| Uterus | 18.7 ± 1.6 |
| Lungs | 18.4 ± 2.5 |
| Ovaries | 17.8 ± 1.7 |
| Heart | 17.0 ± 2.9 |
| Upper large intestine | 16.6 ± 1.7 |
| Lower large intestine | 16.4 ± 1.7 |
| Pancreas | 16.4 ± 1.7 |
| Adrenals | 15.9 ± 1.8 |
| Testes | 14.9 ± 0.1 |
| Total body | 14.1 ± 1.5 |
| Thymus | 12.5 ± 1.6 |
| Muscle | 12.2 ± 1.3 |
| Thyroid | 11.5 ± 0.9 |
| Breasts | 10.1 ± 1.2 |
| Skin | 9.4 ± 1.0 |
| Effective dose | 20.5 ± 2.1 |

Data are presented as mean ± SD
